# Ground-breaking change to the mental health section of the WHO Model List of Essential Medicines: implications for low- and middle-income countries

**DOI:** 10.1017/S2045796024000040

**Published:** 2024-02-01

**Authors:** Corrado Barbui, Davide Papola, Beatrice Todesco, Chiara Gastaldon, Giovanni Ostuzzi

**Affiliations:** 1 WHO Collaborating Centre for Research and Training in Mental Health and Service Evaluation, Department of Neuroscience, Biomedicine and Movement Sciences, Section of Psychiatry, University of Verona, Verona, Italy; 2 Department of Global Health and Social Medicine, Harvard Medical School, Boston, MA, USA

## Introduction

Rational selection of medicines refers to the careful selection, at the country level, of medicines based on the best available evidence to inform practice and to ensure the economic viability of healthcare systems (Barbui *et al.*, 2016). Rational selection facilitates the bulk purchase and easier management of the storage and distribution of medicines and is considered to be a prerequisite for establishing a sustainable supply system or a sound insurance reimbursement system. It also facilitates monitoring of the use of medicines and their quality (World Health Organization, 2017a).

For mental disorders, the rational selection of medicines is particularly relevant. Some of the available psychotropic medicines are duplicative or nonessential, being minor variations of originator products with unclear therapeutic advantages over other medicines already on the market (Barbui and Bighelli, 2013a, 2013b; Barbui and Purgato, 2014). In many cases, new medicines are released with limited information on comparative efficacy and tolerability, leading to uncertainty about their effectiveness compared with other medicines already in use (Barbui and Bighelli, 2013a, 2013b; Erhel *et al.*, 2020). Moreover, newer psychotropic medicines may be considerably more expensive than older medicines (Garattini and Bertele’, 2005).

Aiming to support, at the country level, the rational selection of medicines, since 1977, an essential medicine list (EML) has been drawn up by the World Health Organization (WHO) (World Health Organization, 2002). Essential medicines are expected to be selected by countries and included in national formularies to be available for free, or at affordable prices, to those in need. This is particularly important in low- and middle-income countries (LMICs), where the gap between prevalence and treatment remains unacceptably large (World Health Organization, 2017a, 2022). In these settings, alignment of national formularies with the WHO EML is considered a first, crucial public health step to improve global access to mental healthcare.

The WHO EML undergoes biennial updates, incorporating evidence-based applications rigorously assessed by an independent expert committee appointed by the WHO. For consideration, applications must summarise the available clinical evidence substantiating the comparative effectiveness of the proposed medicine for a specific indication. A summary of data regarding comparative cost and cost-effectiveness is also essential. A structured template is available on the WHO website to enhance application consistency and quality. After decades characterized by minimal updates to the mental health section of the WHO EML, a thorough revision of the whole section has recently been completed by the WHO on the basis of a series of evidence-based applications (Papola *et al.*, 2023) Here, we present the updated mental health section of the WHO EML, and we discuss the expected implications for LMICs in view of the differential availability potential of essential medicines for mental disorders.

## Psychotropic medicines on the 23rd WHO Essential Medicine List

The new mental health section of the WHO EML is presented in Table 1, which reports the medicines with indications for mental disorders, the year of first introduction on the WHO EML and its evolution over the years since 1977. The updated WHO EML includes twenty-three essential medicines for mental disorders, ten of which are antipsychotics (six second-generation and four first-generation antipsychotics), eight are antidepressants (six selective-serotonin reuptake inhibitors and two tricyclic antidepressants), three are mood stabilizers and two are benzodiazepines (Table 1). Essential psychotropic medicines are listed for five priority mental disorders, namely psychotic, depressive, bipolar, anxiety and obsessive-compulsive disorders.

**Table 1.** Medicines with an indication for mental disorders on the 23rd WHO Essential Medicine List

Indication	Formulation	Medicine	Dose	Evolution of the WHO EML (World Health Organization, 2019)
Psychotic disorders	Tablet	□ Haloperidol Therapeutic alternatives - Chlorpromazine	2 mg; 5 mg	Haloperidol and chlorpromazine were included in the first WHO EML in 1977, and a square box was added to both medicines in 1983. The square box of chlorpromazine was removed in 2023 on the basis of a memorandum submitted by the Department of Mental Health and Substance Use of the WHO.
		□ Risperidone Therapeutic alternatives - Aripiprazole - Olanzapine - Paliperidone - Quetiapine	0.25–6.0 mg	Risperidone was added in 2013 on the basis of two applications, one from the Mount Sinai School of Medicine and another from the Massachusetts General Hospital. A square box was added to risperidone in 2023 on the basis of an application from the University of Verona that indicated a selection of second-generation antipsychotics as an alternative to risperidone.
		Clozapine (complementary list)	25–200 mg	Clozapine was added to the complementary list in 2013 based on an application submitted by the Department of Mental Health and Substance Use of the WHO. Inclusion in the complementary list implies that clozapine is indicated for individuals with psychosis who do not respond to other antipsychotics, provided that laboratory facilities are available for regular monitoring of white blood cells.
	Injection (immediate release)	Haloperidol	5 mg in 1 mL ampoule	This formulation of haloperidol was added in 1983.
		Olanzapine	10 mg in vial	This formulation of olanzapine was added in 2023 on the basis of an application from the University of Verona that indicated that chlorpromazine IM injections should be replaced by olanzapine IM injections.
	Injection (long-acting)	□ Fluphenazine Therapeutic alternatives - Haloperidol - Zuclopenthixol	25 mg (decanoate or enantate) in 1 mL ampoule	Fluphenazine long-acting was included in the first WHO EML in 1977. A square box was added in 1983. In 2023, a memorandum from the Department of Mental Health and Substance Use of the WHO indicated a selection of first-generation long-acting antipsychotics as an alternative to fluphenazine long-acting.
		□ Paliperidone Therapeutic alternatives - Risperidone	25 mg; 50 mg; 75 mg; 100 mg; 150 mg (as palmitate) in pre-filled syringe	Paliperidone long-acting was added in 2021 on the basis of an application submitted by the University of Verona that indicated similar effectiveness and safety of first- and second-generation antipsychotic medicines. The WHO Expert Committee decided to include paliperidone long-acting, and risperidone long-acting as an alternative, on the basis of its efficacy, adverse effects, availability and cost.
Depressive disorders	Tablet	Amitriptyline	25 mg; 75 mg	Amitriptyline was included in the first WHO EML, and a square box was added in 1983 to indicate any medicines of the tricyclic antidepressant group. In 2023, based on a memorandum submitted by the WHO Department of Mental Health and Substance Use, the WHO Expert Committee removed the square box, indicating amitriptyline as the only essential tricyclic antidepressant for depressive disorders.
		□ Fluoxetine Therapeutic alternatives - Citalopram - Escitalopram - Fluvoxamine - Paroxetine - Sertraline	20 mg	Fluoxetine was added in 2007 based on an application from the University of Verona. In 2019, a square box was added to fluoxetine, on the basis of a new application jointly submitted by Harvard Medical School and the University of Verona. The expert committee recognized that the inclusion of different antidepressants as essential medicines may be beneficial at the country level to expand therapeutic alternatives for patients and support better procurement. In 2021, the WHO Expert Committee listed a selection of therapeutic alternatives to fluoxetine.
Bipolar disorders	Tablet	Carbamazepine	100 mg; 200 mg; 400 mg	Carbamazepine was included in the first WHO EML in 1977.
		Lithium carbonate	300 mg	Lithium carbonate was included in the first WHO EML in 1977.
		Valproic acid	200 mg; 500 mg	Valproic acid was included in the WHO EML in 1997. A cautionary note was added in 2021 about its use in pregnancy and in women and girls of child-bearing potential.
		□ Quetiapine Therapeutic alternatives - Aripiprazole - Olanzapine - Paliperidone	25 mg; 100 mg; 150 mg; 200 mg; 300 mg (immediate release); 50 mg; 150 mg; 200 mg; 300 mg; 400 mg (modified release)	Second-generation antipsychotics for the pharmacological treatment of bipolar disorders were added to the WHO EML in 2023, based on an application submitted by the University of Verona. A square box was added to quetiapine indicating a selection of second-generation antipsychotics as an alternative to quetiapine.
Anxiety disorders	Tablet	□ Diazepam Therapeutic alternatives - Lorazepam	2 mg; 5 mg.	Diazepam was included in the first WHO EML in 1977, and a square box was added in 1983. In 2023, based on an application submitted by the University of Verona, a cautionary note was added to indicate short term use only, and lorazepam was selected as an alternative to diazepam.
		□ Fluoxetine Therapeutic alternatives - Citalopram - Escitalopram - Fluvoxamine - Paroxetine - Sertraline	20 mg	Considering the lack of antidepressants for generalized anxiety disorder, panic disorder and social anxiety disorder in the WHO EML, in 2023, based on an application submitted by the University of Verona, fluoxetine with a square box indicating a selection of alternative antidepressants was added.
Obsessive-compulsive disorders	Tablet	Clomipramine	10 mg; 25 mg	Clomipramine was included in the WHO EML in 1993.
		□ Fluoxetine Therapeutic alternatives - Citalopram - Escitalopram - Fluvoxamine - Paroxetine - Sertraline	20 mg	Considering the lack of second-generation antidepressants for obsessive-compulsive disorders, in 2023, based on an application submitted by the University of Verona, fluoxetine with a square box indicating a selection of alternative antidepressants was added.

The revision determined an increase in the overall number of medicines considered essential for mental disorders, with a balance between old and new medicines: some widely available and affordable medicines are still on the list after more than 45 years (e.g., haloperidol, chlorpromazine, fluphenazine long-acting, amitriptyline, lithium, carbamazepine and diazepam), together with others recently added to represent current best evidence-based choices. Notably, for antipsychotics, this balance refers also to short- and long-acting intramuscular formulations, with the presence of short-acting olanzapine and long-acting paliperidone (1-month formulation) and risperidone (Table 1) (Ostuzzi *et al.*, 2022).

In the updated WHO EML, some psychotropic medicines are considered essential for different conditions. For example, aripiprazole, olanzapine, paliperidone and quetiapine are listed as essential medicines for both psychotic and bipolar disorders, and selective serotonin uptake inhibitors are listed as essential for depressive, anxiety and obsessive-compulsive disorders. This approach allowed us to keep a balance between the need to list a relatively low number of medicines, in agreement with the concept of being essential, and the pressing need to align the WHO EML with the current best international and national standards on the pharmacological treatment of mental disorders. The new WHO EML is this way reinforced in its role of being a reference model list for countries that want to implement evidence-based choices and not only for countries that want to select the most affordable medicines.

In the new WHO EML, all psychotropic medicines with a ‘square box’ have a restricted list of specific alternative medicines that are considered therapeutic equivalents, as opposed to previous versions of the list which included several ‘unrestricted’ square boxes, implying that any medicines of the same pharmacological class could be considered as effective therapeutic alternatives (Cappello *et al.*, 2020). This change increases the focus of the list, as now each therapeutic alternative is named and gives more value to the background evidence for each medicine rather than making general assumptions about the therapeutic equivalence of medicines in the same pharmacological class.

In line with WHO recommendations of considering pharmacological treatment for mental disorders only in adolescents from 13 years of age (World Health Organization, 2016), haloperidol, chlorpromazine and fluoxetine were removed from the children WHO EML. Notably, this implies that there are no more essential psychotropic medicines for children up to 12 years of age.

The WHO Expert Committee did not accept the recommendation of adding phenelzine for treatment-resistant depression and paliperidone 3-month long-acting intramuscular injections. For phenelzine, it was argued that the evidence of efficacy in treatment-resistant depression is almost absent, and that there are safety issues requiring specialist expert knowledge. Additionally, the use of phenelzine requires adherence to certain dietary requirements, and coadministration of some medicines should be avoided. For these reasons, this medicine is usually prescribed in specialized settings. For paliperidone 3-month long-acting intramuscular injections, the WHO Committee noted that it is not recommended to initiate treatment with the 3-month formulation, rather it is used in patients who demonstrate benefit and tolerance to the 1-month formulation over at least four months. In addition, there is no clear evidence that this formulation is cost-effective compared to paliperidone 1-month long-acting injections, which is already on the list, and available as a generic.

## Availability potential of essential psychotropic medicines

At the country level, using the WHO EML as a reference guide for psychotropic medicine selection should contemplate the differential availability potential of the medicines on the list. Availability pertains to the obtainability of medicines in the public and private sectors (Bigdeli *et al.*, 2013). At the country level, availability is determined by processes for medicine regulation and functional supply systems, including procurement and distribution chains. Notably, some medicine characteristics may facilitate or hamper availability, depending on each country's organization of healthcare. Borrowing the taxonomy of elements that make psychosocial interventions scalable in LMICs, that is being transdiagnostic, task-shifting and low-resource intensity (World Health Organization, 2017b), the country level availability potential of the medicines included in the revised mental health section of the WHO EML may be outlined, as suggested in Table 2.

**Table 2.** Availability potential of medicines for mental disorders included on the 23rd WHO Essential Medicine List

Psychotropic medicine	Trans-diagnostic	Task-shifting	Non-medical IP or CP	Monitoring	Under International Control	Median buyer price (USD)	Median supplier price (USD)
Aripiprazole	Partially	Yes	CP	Medium-intensity	No	N/A	N/A
Chlorpromazine	Yes	Yes	CP	Medium-intensity	No	0.0104/tab (25 mg)	0.0081/tab (25 mg)
Clozapine	No	No	None	High-intensity	No	0.1760/tab (25 mg)	N/A
Fluphenazine	Yes	Yes	CP	Medium-intensity	No	0.9581/ml	0.8340/ml
Haloperidol	Yes	Yes	CP	Medium-intensity	No	0.0669/tab (5 mg)	0.0092/tab (5 mg)
Olanzapine	Partially	Yes	CP	Medium-intensity	No	0.0937/tab (5 mg)	N/A
Paliperidone	Partially	Yes	CP	Medium-intensity	No	N/A	N/A
Perphenazine	Yes	Yes	CP	Medium-intensity	No	N/A	N/A
Quetiapine	Partially	Yes	CP	Medium-intensity	No	N/A	N/A
Risperidone	Partially	Yes	CP	Medium-intensity	No	0.0204/tab (2 mg)	0.0714/tab (2 mg)
Amitriptyline	No	Yes	IP	Low-intensity	No	0.0281/tab (25 mg)	0.0084/tab (25 mg)
Citalopram	Partially	Yes	IP	Low-intensity	No	N/A	N/A
Clomipramine	Partially	Yes	IP	Low-intensity	No	0.0477/tab (25 mg)	0.0358/tab (25 mg)
Escitalopram	Partially	Yes	IP	Low-intensity	No	N/A	N/A
Fluvoxamine	Partially	Yes	IP	Low-intensity	No	N/A	N/A
Fluoxetine	Partially	Yes	IP	Low-intensity	No	0.0103/tab (20 mg)	0.0425/tab (20 mg)
Paroxetine	Partially	Yes	IP	Low-intensity	No	N/A	N/A
Sertraline	Partially	Yes	IP	Low-intensity	No	0.0234/tab (50 mg)	N/A
Carbamazepine	No	Yes	CP	Medium-intensity	No	0.0202/tab (200 mg)	0.0185/tab (200 mg)
Lithium carbonate	No	No	None	High-intensity	No	N/A	N/A
Valproic acid	No	Yes	CP	Medium-intensity	No	N/A	0.1319/tab (500 mg)
Diazepam	Yes	Yes	IP	Low-intensity	Yes	0.0113/tab (5 mg)	0.0096/tab (5 mg)
Lorazepam	Yes	Yes	IP	Low-intensity	Yes	0.0266/tab (2 mg)	N/A

Trans-diagnostic: Yes = medicines can be used to target specific symptoms occurring in different mental disorders; No = medicines can be used for a specific diagnosis or indication or label; Partially = medicines can be used in two or more mental disorders.

Task-shifting: Yes = medicines can be used by non-specialist healthcare providers who are trained to prescribe pharmacological treatments; No = medicines can be used by non-specialist healthcare providers who are trained to prescribe pharmacological treatments only in consultation with a specialist or under the supervision of a specialist.

IP = initial prescribing; CP = continued prescribing only.

Monitoring: High-intensity = regular clinical, laboratory and ECG monitoring required; medium-intensity = regular clinical, laboratory and ECG monitoring suggested; low-intensity = regular clinical, laboratory and ECG monitoring as needed.

Buyer price: Price available to organizations conducting the tender or procurement, e.g. South Africa Department of Health, Peru Department of Health, Organization for Eastern Caribbean Procurement Services, etc.; supplier price: price available to organizations who maintain a warehouse and supply items directly to customers, e.g. UNICEF supply division, United Nation population fund, Affordable medicines for Africa, etc. *Source:* MSH International medical products price guide: https://mshpriceguide.org/en/home/ (year 2015).

First, some essential psychotropic medicines can be considered transdiagnostic, implying that they can be used to target specific symptoms across disorders rather than specific diagnostic entities, such as, for example, benzodiazepines and first-generation antipsychotics. By contrast, other medicines can be used for a specific diagnosis or indication or label, such as clozapine, lithium or carbamazepine. Some other medicines may be considered partially transdiagnostic, such as second-generation antipsychotics or antidepressants, as they can be used for different mental disorders (Table 2). At the country level, the medicine selection process should carefully consider these differences, as they would imply, for the selected medicines, specific regulatory decisions which, in turn, may strongly affect availability, such as, for example, labelling some medicines for use in individuals with specific diagnoses versus specific symptoms (Barbui *et al.*, 2017).

Second, almost all essential psychotropic medicines on the WHO EML might be subject to a task-shifting delivering modality; that is, they might be prescribed by non-specialist healthcare providers who are trained to prescribe pharmacological treatments. This position is already supported by several organizations, including the WHO, which advocates the prescription of psychotropic medicines by non-specialists, except clozapine and lithium (World Health Organization, 2016). These two medicines are generally recommended to be used by non-specialist healthcare providers only in consultation with a specialist or under the supervision of a specialist (Table 2). Notably, clozapine and lithium would also need a medical doctor to issue initial and continued prescriptions. Depending on country regulations, the same may not apply to other essential psychotropic medicines, which may be prescribed by trained non-medical staff, for example antidepressants and benzodiazepines (Maier, 2019). For some other medicines, initial prescribing may be up to a medical doctor, with continued prescribing up to non-medical staff, say for example antipsychotics or mood stabilizers (Table 2). At the country level, these prescribing considerations should inform the selection process, as they imply, for the selected medicines, regulatory decisions on which medicines may be prescribed by doctors only or also by other professionals, including initial and subsequent prescriptions, and a decision on the level of the healthcare system where each essential psychotropic medicine should be available.

Third, some essential psychotropic medicines require specific and high-intensity monitoring, for example clozapine and lithium, and this may decrease their availability potential, while other medicines are generally prescribed alongside regular clinical, laboratory and ECG monitoring, for example antipsychotics and mood stabilizers, which may also be an obstacle to availability in some settings (Table 2). Among essential psychotropic medicines, antidepressants and benzodiazepines usually need clinical, laboratory and ECG monitoring on a less intense basis (Table 2). As for the previous considerations, at the country level, the selection process should take these monitoring requirements into due consideration, as they imply, for the selected medicines, specific regulatory decisions on monitoring requirements affecting availability to the end-users.

Fourth, at the country level, the selection process is informed by the cost of medicines, which is reported in Table 2 for essential psychotropic medicines. The table presents the buyer price, that is the price available to organizations conducting the tender or procurement (typically countries), and the supplier price, that is the price available to organizations who maintain a warehouse and supply items directly to customers (i.e., international organizations doing fieldwork). For both indicators, we note that, for most second-generation antipsychotics and several selective serotonin reuptake inhibitors, the price was not available, which may indicate a price and affordability barrier. Likely, the fact that these medicines are now featured in the WHO EML will activate a process for their inclusion among those available for purchase internationally, with buyer and supply prices available. This inclusion may boost their purchase, which, in turn, should eventually bring down prices and increase the likelihood that they will be included in universal healthcare packages. Moreover, public health organizations such as the United Nations’ Medicines Patent Pool might negotiate licensing agreements with pharmaceutical companies to manufacture competitive generics of some of the included medicines (Burrone *et al.*, 2019).

Taking into consideration, the availability potential of psychotropic medicines during the selection process may be relevant given existing evidence showing that, while alignment of the WHO EML with national formularies is substantial for psychotropic medicines (Todesco *et al.*, 2023), the availability of the selected medicines is rather poor at the country level, especially in LMICs (Shi *et al.*, 2023; Todesco *et al.*, 2022). Decision-makers could, therefore, consider the potential availability of psychotropic medicines as a factor informing the selection process, aiming to select from the WHO EML the medicines that are more likely to be widely available in relation to the characteristics of each country's healthcare system organization and development.

In conclusion, the updated WHO EML provides an exceptional chance to enhance the selection of the safest and most effective psychotropic medicines at the country level. It may also facilitate the implementation of regulatory policies that enhance their accessibility. These endeavours can significantly promote global mental health equity and expand universal health coverage. Ultimately, this should lead to the creation of a more inclusive and equitable mental healthcare system worldwide.
